# British Hospitals Association

**Published:** 1921-03-26

**Authors:** 


### British Hospitals Association.

### Kent Regional Committee Formed.

A meeting convened by the Kent and Canterbury Hospital was held at Canterbury on March 8, for the purpose of forming a Regional Committee of the British Hospitals Association. The Conference was open to all hospitals in Kent, and was well attended. The chair was taken by the Very Rev. the Dean of Canterbury (Dr. Wace), Chairman of the Kent and Canterbury Hospital, who was immediately supported by the Hon. Henry Hannen, Chairman of the West Kent General Hospital, Maidstone.

After an interesting discussion it was unanimously decided to form a Kent Regional Committee of the British Hospitals Association. As the area thus covered would be a large one, and the means of transit not very convenient, it was further decided to form two sections of the Committee, one for East Kent and one for West Kent. Mr. F. P. Carroll, Secretary of the Kent and Canterbury Hospital, and Mr. H. St. John Wood, Secretary of the West Kent General Hospital, Maidstone, were appointed the respective Hon. Secretaries. The Conference agreed that the sections should deliberate separately, but that they should meet together for the purpose of adopting resolutions in the name of the Kent Regional Committee. The next meeting of the full Committee will be held at Ashford on April 16, when representatives will be appointed to appear before Lord Cave's Committee of Inquiry.

